# New COI-COII mtDNA Region Haplotypes in the Endemic Honey Bees *Apis mellifera intermissa* and *Apis mellifera sahariensis* (Hymenoptera: Apidae) in Algeria

**DOI:** 10.3390/insects15070549

**Published:** 2024-07-20

**Authors:** Amira Chibani Bahi Amar, Nacera Tabet Aoul, Riad Fridi, Alain Vignal, Kamila Canale-Tabet

**Affiliations:** 1 Laboratoire de Génétique Moléculaire et Cellulaire (LGMC), Département de Génétique Moléculaire Appliquée, Université des Sciences et de la Technologie d’Oran Mohamed Boudiaf, USTOMB, BP 1505, El M’naouer, Oran 31000, Algeria; 2 Department of Biotechnology, Faculty SNV, University of Oran1 Ahmed Ben Bella, Oran 31000, Algeria; 3 GenPhySE, Université de Toulouse, INRAE, INPT, INP-ENVT, 31326 Castanet-Tolosan, France

**Keywords:** mitochondrial DNA, COI-COII intergenic region, *Apis mellifera intermissa*, *Apis mellifera sahariensis*, A lineage haplotypes, sequencing, Algeria

## Abstract

**Simple Summary:**

Currently, one of the most raised issues regarding the preservation of endemic honey bees is the importation of foreign subspecies, leading to genetic introgression and hybridization between subspecies. In Algeria, many beekeepers have reported importing foreign honey bees, notably the Italian *Apis mellifera ligustica*, for their high honey production. In order to provide a molecular characterization and a better knowledge of the genetic background of the Algerian honey bees subspecies *A. m. intermissa* and *A. m. sahariensis*, we analyzed the widely used COI-COII mitochondrial marker. In this study, we sequenced 139 drones of which 68 were from 20 regions in Algeria, and 71 individuals as a reference population, sampled in Europe, the South West Indian Ocean archipelagos, and Madagascar, representing the evolutionary lineages M, C, and A. As a result, we found that all Algerian samples belong to the African lineage A with the identification of 24 haplotypes, of which 16 are reported for the first time. The reference population shows ten different haplotypes distributed over the three lineages, with the identification of a new haplotype carried by two individuals sampled in France. The results of this survey show the absence of evidence of genetic introgression between Algerian and European honey bees.

**Abstract:**

The practice of beekeeping in Algeria is of great cultural, social, and economic importance. However, the importation of non-local subspecies reported by beekeepers has disrupted the natural geographical distribution area and the genetic diversity of the native honey bees. To assess the genetic diversity of *A. m. intermissa* and *A. m. sahariensis*, and their relationships with African and European subspecies, the COI-COII intergenic region was analyzed in 335 individuals, 68 sampled in Algeria, 71 in Europe, Madagascar, and the South West Indian Ocean archipelagos, and 196 sequences recovered from GenBank. The results show the presence of the A lineage exclusively in Algerian samples with the identification of 24 haplotypes of which 16 are described for the first time. These haplotypes were found to be shared by both subspecies, with A74 being the most common haplotype in the population studied. The sequence comparison indicates the existence of three polymorphisms of the COI-COII marker: P0Q, P0QQ, and P0QQQ. One new haplotype was identified in the M lineage in samples from France. No evidence of genetic introgression within the Algerian honey bee population was detected. These data enhance our knowledge of the genetic diversity and emphasize the importance of protecting these local subspecies.

## 1. Introduction

The western honey bee (*Apis mellifera*) is among the most effective pollinators, playing an important ecological and economical role in ecosystems and agriculture globally [1]. Diversity within the *Apis mellifera* species has been studied from various angles, including genetics, morphological, ecological, and behavioral traits. Historically, *Apis mellifera* has about 30 recognized subspecies [2] divided into four main evolutionary lineages, according to their geographical range: A: African; M: Northern and Western European; C: Southeastern European; and O: Middle Eastern [3,4]. A fifth group has been found in the southern part of the Middle East: Y, which has been described as different from the O lineage found in the north [5]. Previous studies on Algerian honey bees have demonstrated their belonging to the African evolutionary lineage [6,7,8].

The honey bees *Apis mellifera intermissa* [9] and *Apis mellifera sahariensis* [10] are subspecies of the western honey bee *Apis mellifera* and are endemic to Algeria [3]. *A. m. intermissa* is commonly known as the Tellian honey bee and is found in Northern Africa, extending over Algeria, Tunisia, and Morocco [3]. Multiple analyses describe *A. m. intermissa* as a black-colored, fairly aggressive, highly productive honey bee and well adapted to the climate of the region [11,12]. In contrast, the Saharan honey bee *A. m. sahariensis* is found only in the southern part of Algeria and extends to Morocco [10]. It is described as a yellow-colored honey bee and less aggressive than *A. m. intermissa*. Its morphological and behavioral characteristics enable it to thrive in difficult environmental conditions such as high temperatures, drought, and limited water availability [13,14]. Despite this, these subspecies, especially *A. m. sahariensis,* are facing multiple challenges, such as global warming, exposure to pesticides, and certain beekeeping practices in particular transhumance that jeopardize the survival of these honey bees [15].

Mitochondrial DNA (mtDNA) is an extensively used tool to assess genetic diversity and phylogeography among *Apis mellifera* subspecies, especially the hypervariable tRNA^leu^-COII or COI-COII region, having insertion–deletion and copy-number variation polymorphisms in addition to single nucleotide polymorphisms (SNPs) [16,17]. The DraI test has been used on a large scale and has proved its effectiveness in providing valuable insights on population structure, genetic variation, and evolutionary relationships within honey bee populations [5,18,19,20]. The DraI test consists of performing an enzymatic digestion, also known as PCR–RFLP (Restriction Fragment Length Polymorphism), on the non-coding intergenic region of mtDNA, located between the end of the cytochrome c oxidase subunit I gene and the beginning of the cytochrome c oxidase subunit II gene [21]. Variations in restriction patterns reflect the genetic diversity and evolution within *Apis mellifera* subspecies. This region shows differences in nucleotide composition, sequence length, and structural organization between *Apis mellifera* subspecies [22]. The African and European lineages show variation in sequence size, due to the presence, or absence, and number of repeats of the P and Q units, respectively. The African A lineage can be represented by P0 or P1 units, while the M lineage features only the P unit. On the other hand, the European C lineage has no P unit [7]. Multiple studies using mtDNA were conducted to analyze the genetic structure and to assess the genetic diversity and phylogenetic relationships of African honey bee populations [5,23,24].

In order to gain a better understanding of the biogeography of the western honey bees in Algeria, we analyzed the mtDNA haplotype diversity of honey bee samples from northern and southern Algeria. A reference population of previously described samples representing the main evolutionary lineages was used to provide a broader perspective of Algerian honey bees’ genetic diversity in an extended geographical scope. This study aims to (i) contribute to increase our knowledge of the genetic architecture of the mtDNA intergenic region COI-COII in Algerian honey bees, (ii) identify traces of genetic introgression into local honey bee populations in the area, and (iii) describe the mitochondrial genetic diversity of *A. m. intermissa* and *A. m. sahariensis*.

## 2. Materials and Methods

### 2.1. Honey Bee Sampling

**Figure 1:**
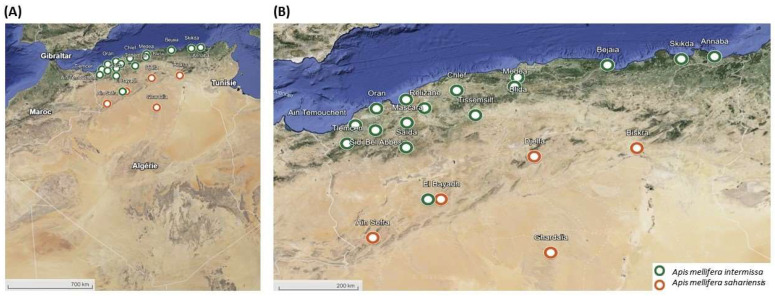
(**A**) Sampling sites of *Apis mellifera intermissa* and *Apis mellifera sahariensis* in 20 regions in Algeria. (**B**) Zoom on sampling areas. Sample sizes for each sampling area are giving in (Supplementary Table S1).

Sampling of haploid drones was conducted in ten regions of Algeria from 2021 to 2022. In total, 60 drones either in the pupal or larval stage were sampled from frames in beehives. This allows the identification of the beehives and ensures each selected drone for the study represents a different colony. In addition, we included 20 adult samples originating from ten different sites in Algeria obtained from a previous study [25] (Figure 1). During the sampling, the distinction between the two subspecies was based on the coloration observed in honey bees. *A. m. intermissa* is known to be a black bee, found in the north of Algeria [9], and *A. m. sahariensis* is described to be a yellow honey bee found in the south of Algeria [10]. Out of the 80 samples, we collected *A. m. intermissa* drones (*n* = 66) at 16 regions in the north, *A. m. sahariensis* drones (*n* = 14) over 5 regions in the south, and we were able to sample both *A. m. intermissa* and *A. m. sahariensis* in 1 common region (EL-Bayadh) (Supplementary Table S1).

In addition, 75 samples representing the three evolutionary lineages A, C, and M were obtained from INRAE Occitanie—Toulouse, France. The African (A) lineage was represented by *A. m. unicolor* individuals from the South West Indian Ocean archipelagos (SWIO) and Madagascar. In Europe, the honey bees *A. m. mellifera*, *A. m. carnica*, *A. m. ligustica*, *A. m. caucasia,* and Buckfast were represented, originating from five countries: France, Italy, Germany, Poland, and Slovenia (Supplementary Table S1).

### 2.2. DNA Extraction

The haploid drone samples were stored in absolute ethanol at −20 °C until molecular analysis. Total DNA was extracted from the head and thorax for adult samples, while the whole body was used for larval or pupal samples. The extraction was carried out as follows: we added 490 µL cell lysis buffer (TNES-Urea: 1 M Tris HCl, pH 8, 0.5 M EDTA, 3 M NaCl, 10% SDS) to the material, then two volumes of Proteinase K (eurobio GEXPRO01) 10 mg/mL in two steps: (i) 12.5 µL with incubation during 3 h at 56 °C; (ii) 5 µL with overnight incubation at 37 °C. Cell debris precipitation was realized by adding 200 µL of 3 M NaCl to samples, then a centrifugation at 12,500× *g* at 4 °C for 30 min was performed. The supernatant was transferred to new tubes with 2.5 µL of RNase (100 mg/mL) and precipitation of DNA was carried out by adding absolute ethanol (100%). Each DNA pellet was rinsed in 70% ethanol, air dried, resuspended in 100 µL TE buffer 10/0.1 (10 mM Tris HCl, 0.1 mM EDTA), and incubated overnight at 37 °C under constant agitation. DNA quality and quantity assessment were carried out by dosing methods: NanoDrop 8000 spectrophotometer (Thermo Scientific, Wilmington, DE, USA), PicoGreen fluorescence assay, and electrophoresis in a 0.8% agarose gel.

### 2.3. Mitochondrial DNA Analysis

The PCR amplification of the COI-COII intergenic region was carried out using E2 (5′-GGCAGAATAAGTGCATTG-3′) and H2 (5′-CAATATCATTGATGACC-3′) primers following the method and reaction conditions as described in Garnery et al., 1993 [21]. A volume of 2.5 µL from each PCR product was examined on a 2% agarose gel.

The sequencing reaction was prepared as follows: 2.5 µL of each PCR product was purified by adding 1 µL of each exonuclease and TSAP (Shrimp Alkaline Phosphatase) in an 18 µL final volume. The sequencing reaction mixture was split into two volumes, and 1 µL of each primer (forward and reverse) was added. All PCR products were sent to GENEWIZ (Azenta Life Sciences, Leipzig, Germany) for Sanger sequencing in both directions. The 310 sequences produced (corresponding to 155 individuals) were manually checked for base calling using ChromasPro 2.1.10.1. (Technelysium Pty Ltd., Tewantin, Australia), and sequences with bad quality were removed. The 139 sequences retained were aligned with 196 published sequences available in GenBank (https://www.ncbi.nlm.nih.gov/genbank/ accessed on 9 February 2023) using Jalview 2.11.3.3 [26]. Sequences were aligned according to the size and structure of the COI-COII intergenic region.

Additionally, the sequences obtained were digested in silico using the REBASE tool version 212, which simulates DNA digestion by using computational tools supplied by the REBASE database (http://rebase.neb.com/rebase/rebtools.html (accessed on 15 September 2023).

### 2.4. Nomenclature of Novel Haplotypes

The extensive use of the intergenic region COI-COII to analyze the genetic diversity of populations has generated a vast amount of data and sequences, with identical sequences bearing different names and, conversely, different sequences published under the same names. This makes the use of published sequences a real challenge. In this study, new haplotypes were identified and named following the nomenclature system described in Rortais et al. in 2011 [27] and Chavez-Galarza et al. in 2017 [7].

### 2.5. Phylogenetic Analyses

Sequence alignment of the dataset (196 sequences retrieved from GenBank and 139 newly produced), fasta files generation, and phylogenetic tree construction were conducted using MEGA X 11.0.13 [28]. Haplotypes were identified using the package Sidier (version 4.1.0) in R studio. Haplotype median-joining networks were constructed using PopArt version 1.7 [29]. However, the PopArt software is not appropriate to deal with insertions/deletions (indels) and will mask sites exceeding 5% missing data. This induces the loss of the P element due to its absence in sequences belonging to the C lineage. In addition, the absence of duplications of the Q element in some sequences is interpreted by PopArt as sites containing undefined states and will mask them. To overcome these problems, sequences were modified according to the format accepted by PopArt following previous studies [30,31]. Briefly, sequences were re-coded such that indels were transformed into bases and gaps were changed into “A”, “C”, “G”, or “T” according to the possibilities left at the given position. Due to the complexity of the COI-COII intergenic region and size differences between sequences, haplotype networks were constructed independently for each structure (e.g., P0Q, P0QQ, P0QQQ…) [31].

## 3. Results

In this study, we sequenced and analyzed a total of 155 individuals. We removed 16 sequences due to their bad quality. The remaining 139 sequences correspond to 68 individuals collected from 20 regions in Algeria, and 71 representing the three main evolutionary lineages A, M, and C from Europe, the SWIO, and Madagascar. In addition, 196 sequences (A lineage (*n* = 115), M lineage (*n* = 44), C lineage (*n* = 37)) retrieved from GenBank were added as a reference population (Supplementary Table S2). The evolutionary lineages and haplotypes of honey bees were identified based on the genetic structure of the mitochondrial intergenic region COI-COII as previously reported in several studies [7,21,32,33,34].

### 3.1. Honey Bee Evolutionary Lineage and Haplotype Identification

**Figure 2:**
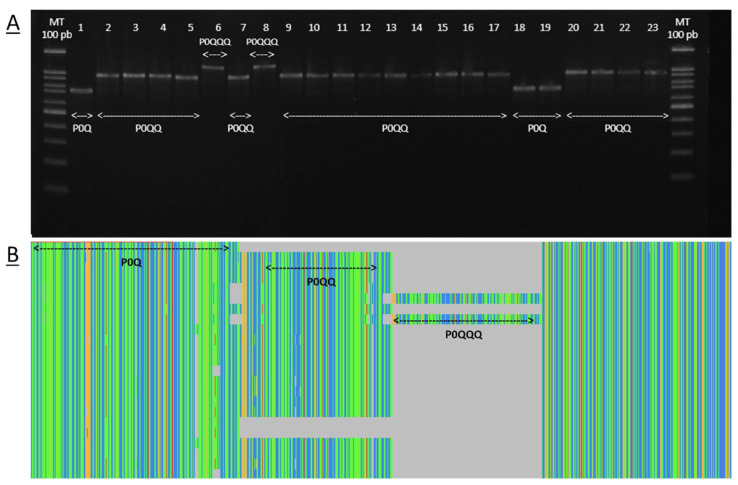
(**A**) PCR products of the mitochondrial COI-COII region, where 1–23 are Algerian samples and MT is a 100 bp ladder. (**B**) Overview from Jalview sequence alignment indicating the variable sites of the mtDNA COI-COII marker; the sequences correspond to the individuals in Figure 2A, displayed in the same order.

The genetic structure of the COI-COII intergenic region in Algerian samples showed the existence of three polymorphisms: (i) P0Q, (ii) P0QQ, and (iii) P0QQQ (Figure 2), with the P0QQ polymorphism being the most prevalent at 61.8% of occurrences (Table 1).

Out of 68 Algerian samples analyzed, a total of 24 haplotypes were identified in this study including 16 haplotypes described for the first time (Table 2). Two haplotypes newly described were found to be very similar to those previously described by Attia et al. in 2023 [35], exhibiting slight variations (one or two nucleotides). In order to standardize the labelling of haplotypes and to avoid redundancy, based on the criteria described previously [7,27], we renamed the haplotypes as follows (nomenclature used in Attia et al., 2023 > nomenclature used in this paper): Alg5 > A71; Alg1 > A74c; Alg2 > A74d; Alg3 > A74e; and Alg4 > A74f.

All haplotypes previously and newly described in Algerian honey bees identified in this study belong to the African lineage A. Furthermore, the genetic structure of the mtDNA intergenic region of the reference samples sequenced in this study showed five different polymorphisms: P0Q, P0QQ, PQ, PQQ, and Q (Table 1), with the identification of 10 different haplotypes distributed over the three lineages A, M, and C (Supplementary Table S3).

### 3.2. COI-COII Haplotypes’ Distribution and Comparison

Among the haplotypes identified in the Algerian honey bee population, the most predominant haplotype is the newly described haplotype A74 (P0QQ), representing 24% of the total sample size in Algerian subspecies, found in both *A. m. intermissa* and *A. m. sahariensis* (Supplementary Table S3), and distributed across seven different regions (Supplementary Table S4). The novel haplotype A1x was found in six different regions in the country (Supplementary Table S4) with a percentage of 10.2% of the Algerian honey bee population, and was shown to exhibit the P0Q structure. The widely known haplotypes of the African lineage (A1n (P0Q), A8d (P0Q), A8f (P0Q)), and previously reported to be present in the North African honey bee population [7], were recorded with an overall percentage of 7.3% for the A1n haplotype and 6% for both A8d and A8f (Supplementary Table S3).

Within the African A lineage, all *A. m. unicolor* individuals analyzed showed a P0Q structure and exhibited exclusively the haplotype A1. Interestingly, one *A. m. mellifera* individual sampled in France clustered with the African lineage, presenting a mitochondrial haplotype A4 and a P0QQ genetic structure. Among the M lineage, three haplotypes were identified: M4 and M6 had been previously reported and the third haplotype M4r was deemed to be a novel haplotype, carried by two *A. m. mellifera* individuals from France with a PQQ structure (Supplementary Table S3). Within the C lineage, six haplotypes were detected in individuals from Europe and SWIO islands, with a predominance of the C1a and C2_d haplotypes shown by 37% and 32% of C lineage individuals, respectively.

### 3.3. Evolutionary Lineages Distribution and Relationships among Haplotypes

**Figure 3:**
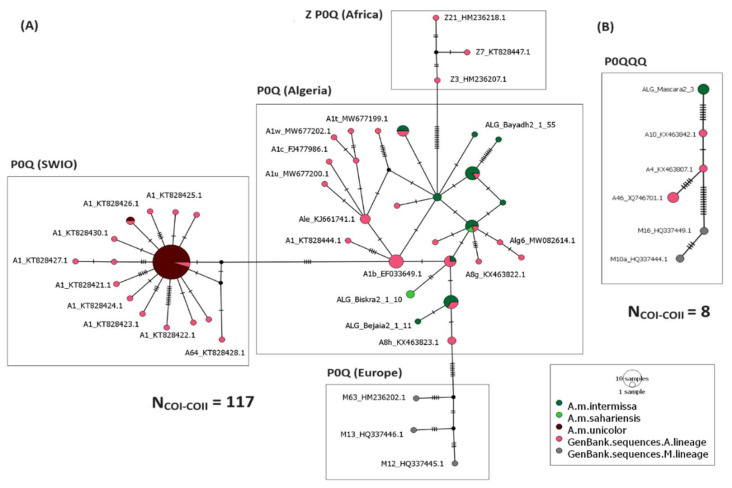
(**A**) Median-joining inferred from individuals with the P0Q intergenic structure. (**B**) Median-joining constructed from individuals with the P0QQQ intergenic structure.

The median-joining network based on the P0Q intergenic structure indicates the presence of four separate groups (Figure 3A). The first group shows sequences of *A. m. intermissa* and *A. m. sahariensis* subspecies from Algeria, newly sequenced and previously published, clustering with sequences from the A lineage retrieved from GenBank, corresponding to *A. m. intermissa*, *A. m. sahariensis,* and *A. m. iberiensis*. The second group presents sequences from the SWIO islands and Madagascar that are grouped with published sequences corresponding to the *A. m. unicolor* subspecies. The third group contains reference sequences from the M lineage, and the fourth group shows sequences corresponding to the African Z sub-lineage subspecies *A. m. lamarckii*. The median-joining network produced from sequences with the P0QQQ intergenic structure shown in Figure 3B illustrates three groups: (i) the Algerian node represented by the newly described A77′ haplotype (individual ALG_Mascara2_3), (ii) the second group of the Iberian honey bee *A. m. iberiensis* sequences representing the A lineage; and (iii) the third group indicating sequences from the M lineage.

**Figure 4:**
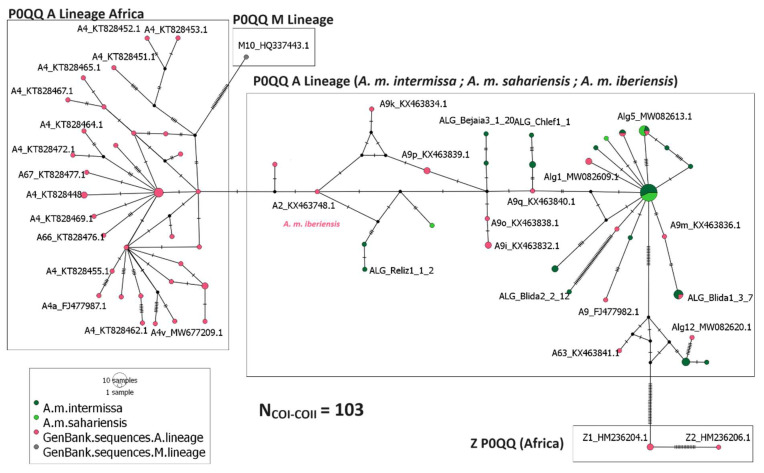
Median-joining network inferred from individuals with the P0QQ intergenic structure.

A total of 103 sequences analyzed in this study presented the P0QQ intergenic structure. The Algerian honey bees *A. m. intermissa* and *A. m. sahariensis* appear to be close to the Iberian honey bee *A. m. iberiensis*. Individuals from the African A lineage form a separated cluster comprising *A. m. unicolor*, *A. m. scutellata,* and *A. m. anadasonii* sequences (Figure 4).

## 4. Discussion

The genetic diversity and population structure of honey bees has been extensively investigated using the mitochondrial non-coding region COI-COII [22]. The results of this study provide a better description of honey bee populations in Algeria. A total of 335 sequences were analyzed, including 139 sequences obtained from Algerian, SWIO, Madagascar, and European samples, and 196 sequences representative of the main evolutionary lineages A, C, and M collected from GenBank.

Sequence analysis of the 68 individuals sampled in Algeria revealed 24 haplotypes, including 16 described for the first time and 8 haplotypes previously published [7,31,34,35]. Our results indicate that the most common haplotypes in Algeria are the novel haplotypes A74 (24%) and A1x (10%), followed by the previously described haplotypes A1n (7.3%), A8d, A8f, and A71 (previously Alg5) (6%), respectively. The haplotypes A8d, A71 (Alg5), A1x, and A74 are found to be shared by the two native subspecies in Algeria (Supplementary Table S3).

The Algerian honey bee population has largely been described based on morphometric observations [11,36,37], microsatellites [20], and mitochondrial DNA [38]. Based on the COI-COII mtDNA marker, Bouzeraa et al. (2020) conducted a study using the PCR-RFLP approach to assess the genetic diversity of Algerian honey bees, and they have demonstrated the difficulties in describing the genetic architecture of the samples and determining with precision their variants (e.g., P0Q or PQ) based solely on the DraI test [38]. These results support the fact that despite the PCR-RFLP DraI method having proven effectiveness in identifying sampled colonies [22], sequencing remains necessary to fully describe the haplotypes and genetic structure of the analyzed individuals. The sequence analysis of the intergenic non-coding region COI-COII in this study shows that the Algerian honey bee population is characterized by three types of intergenic region architecture: (i) P0Q, (ii) P0QQ, and (iii) P0QQQ. These results are concordant with those published previously by Achou et al. 2015 [39]. All Algerian individuals have the P0 form of the P element, which is typical of the African A lineage [5,32,40], but they are differentiated with the number of repetitions of the Q element and by their DraI restriction profiles.

Although *A. m. intermissa* and *A. m. sahariensis* are geographically separated from European honey bee populations, it has been previously reported that European honey bees such as *A. m. ligustica* and Buckfast have been imported by local beekeepers because of their high productivity [20]. Based on the PCR-RFLP DraI test, Achou et al. (2015) reported the presence of foreign honey bees from the M and C lineages in Algeria, representing 3.1% of the population studied. The M lineage was detected in four regions [39], two of which are common to our study (Annaba and Mostaganem) where we did not find any trace of introgression. This can be explained by the different beekeeping practices, in particular the importation of queen bees.

Interestingly, in the 14 *A. m. sahariensis* specimens sampled in our study, two newly described haplotypes (A74b and A75) are found exclusively in this subspecies. The current sample size of 68 individuals is rather small, but by increasing the number of *A. m. sahariensis* and *A. m. intermissa* samples, we will eventually be able to see whether these haplotypes are specific to the Saharan honey bee.

Our data support the hypothesis of the absence of evidence of genetic introgression within Algerian honey bees, as shown in Figure 3 and Figure 4 where all Algerian sequences cluster together and stand apart from individuals from the M and C lineages. These findings align with those reported by a recent study conducted on the analysis of the whole-genome sequence of *A. m. intermissa* and *A. m. sahariensis*, where Algerian and European individuals form two distinct clusters [41]. The closeness observed between Algerian and Iberian honey bees (Figure 4) (Supplementary Figure S2) is due to the African mitochondrial origin of *A. m. iberiensis* individuals from the south of the Iberian Peninsula that are near to the Gibraltar strait [7].

Among the samples analyzed, 32.9% of *A. m. unicolor* subspecies belong the African lineage A and all the sequences correspond to the A1 haplotype, the remaining 7.1% belong to the C lineage and show three haplotypes: the C2 haplotype found on Reunion Island, and the C2_d and C1_a haplotypes found in Rodrigues (Supplementary Figure S1). Our results concerning the native subspecies of SWIO and Madagascar *A. m. unicolor* are fully concordant with those published by Techer et al. (2017) where the A1 haplotype is shared by all the SWIO archipelagos except for Rodrigues [31,42].

The African honey bee populations split into three evolutionary lineages A, O, and Y, comprising at least 11 different subspecies described to date [22,31], with a predominance of the A lineage [5,22,24]. The median-joining networks show a split between Algerian and SWIO honey bee populations, although they both belong to the A lineage (Figure 2 and Figure 3). These results confirm the high mtDNA variability observed within the African lineage. The African honey bee subspecies have been widely described with morphometrics and molecular tools to provide a better knowledge on the diversity and geographical distributions of these subspecies [3,5,10,17,43]. The high diversity observed in African honey bees in morphological, behavioral, and molecular aspects is due to local adaptations to different environmental factors [3,43,44], but also due to the introduction of foreign subspecies which has led to genetic introgression and hybridization between subspecies [24].

Despite the large number of studies conducted on the intergenic mitochondrial region COI-COII in Europe [21,27,40,45], we report for the first time the haplotype M4r—carried by two individuals sampled in France displaying the PQQ intergenic structure. Interestingly, two specimens sampled in France, described earlier as *A. m. mellifera* individuals from the M lineage [46], show type C mitochondria (Q structure) and exhibit the haplotypes C2 and C31. The use of the mitochondrial COI-COII marker is considered as a starting point for analyzing honey bee diversity. Growing evidence on this hypervariable region suggests studies should be extended to other parts of the mitochondrial genome and should be associated as much as possible with nuclear DNA investigations.

## 5. Conclusions

The findings of this study expand our knowledge of the genetic diversity of Algerian honey bee populations. Based on the COI-COII mitochondrial marker, we have identified 16 haplotypes reported for the first time in Algeria and 8 haplotypes previously described, as well as an M lineage haplotype newly described in France. All the individuals sampled in Algeria belong to the African evolutionary lineage. These results support the absence of genetic introgression traces in *A. m. intermissa* and *A. m. sahariensis*. Although multiple studies previously reported exchange with European subspecies, no trace of introgression was detected, at least in our sampling. However, to confirm our results of the absence of introgression and specific *A. m. sahariensis* haplotypes it would be interesting to extend the study to a larger number of individuals in particular *A. m. sahariensis* from different beekeepers and sampling areas.

As a perspective, we aim to analyze the complete mitochondrial genome of Algerian honey bees, in order to provide a current view of the genetic composition of the Algerian native subspecies and to supply molecular tools that can be used in a biodiversity preservation policy of these subspecies.

## Figures and Tables

**Table 1:** Structural organization of the intergenic COI-COII marker in the studied populations.

Sampling Areas	Intergenic Variant	Percentage of Occurrence
Algeria	P0Q	35.3%
	P0QQ	61.7%
	P0QQQ	3%
SWIO and Madagascar	P0Q	92.9%
	Q	7.1%
Europe	P0QQ	3.4%
	PQ	13.8%
	PQQ	27.6%
	Q	55.2%

**Table 2:** New haplotypes identified with their GenBank accession numbers.

L ^1^	GS ^2^	Subspecies	OS ^3^	Cities	Haplotypes	GB Accession ^4^
**A**	P0Q	*intermissa* *sahariensis*	Algeria	Biskra; AinSefra; Bejaia; Chlef; Bayadh; Tissemssilet	A1x	PP667401
**A**	P0Q	*intermissa*	Algeria	Bejaia	A8k	PP667402
**A**	P0QQ	*intermissa*	Algeria	Mascara; Mostaganem; Relizane	A68	PP667403
**A**	P0QQ	*intermissa*	Algeria	Mostaganem	A69	PP667404
**A**	P0QQ	*intermissa*	Algeria	Bejaia	A70	PP667405
**A**	P0QQ	*intermissa*	Algeria	Sidibelabes	A71a	PP667406
**A**	P0QQ	*intermissa*	Algeria	Sidibelabes; Relizane	A72	PP667407
**A**	P0QQ	*intermissa*	Algeria	Chlef; Sidibelabes; Mostaganem	A73	PP667408
**A**	P0QQ	*intermissa*	Algeria	Mascara	A70a	PP667409
**A**	P0QQ	*intermissa*	Algeria	Mascara	A74a	PP667410
**A**	P0QQ	*intermissa*	Algeria	Mostaganem	A73a	PP667411
**A**	P0QQ	*intermissa* *sahariensis*	Algeria	Bayadh; Mostaganem; Tlemcen Oran; Relizane; Ghardaia; Djelfa	A74	PP667412
**A**	P0QQ	*sahariensis*	Algeria	Djelfa	A74b	PP667413
**A**	P0QQ	*sahariensis*	Algeria	AinSefra	A75	PP667414
**A**	P0QQ	*intermissa*	Algeria	Sidibelabes	A76	PP667415
**A**	P0QQQ	*intermissa*	Algeria	Mascara	A77′	PP667416
**M**	PQQ	*mellifera*	France	NA ^5^	M4r	PP667417

^1^ L: Lineages; ^2^ GS: Genetic structure; ^3^ OS: Origin of samples; ^4^ GB Accession: GenBank accession; ^5^ NA: Not available.

## Data Availability

Sequences for haplotypes newly described in this study have been deposited in GenBank under the following accession numbers: PP667401—PP667417.

## Supplementary Materials

The following supporting information can be downloaded at https://www.mdpi.com/article/10.3390/insects15070549/s1, Figure S1: Median-joining network inferred from sequences from C lineage (Q), with the C.lineage.Europe sequences correspond to individuals sampled in Europe with no data reported on the subspecies name; Figure S2. Phylogenetic tree of A lineage haplotypes. The tree is neighbor-joining. Nodes are supported by 1000 bootstraps; Table S1. Sampling sites in Algeria, the South West Indian Ocean archipelagos (SWIO), Madagascar, and Europe. *n* = sample size; Table S2: Description of reference population inferred from NCBI; Table S3: Haplotypes identified with the percentage of occurrence in each sampling areas: Algeria, the South West Indian Ocean archipelagos (SWIO), Madagascar, and Europe. *n* = sample size; Table S4: Haplotype frequencies for sampling sites in Algeria, the SWIO, Madagascar, and Europe. Sample sizes are mentioned in the third line.
