# Supplementary material for: New COI-COII mtDNA Region Haplotypes in the Endemic Honey Bees Apis mellifera intermissa and Apis mellifera sahariensis (Hymenoptera: Apidae) in Algeria
Source: Insects. 2024 Jul 20;15(7):549. doi: 10.3390/insects15070549 (PMC11277165; doi:10.3390/insects15070549)
Supplement: Supplementary file 1 [file insects-15-00549-s001.zip › Supplementary_Figures.pdf]

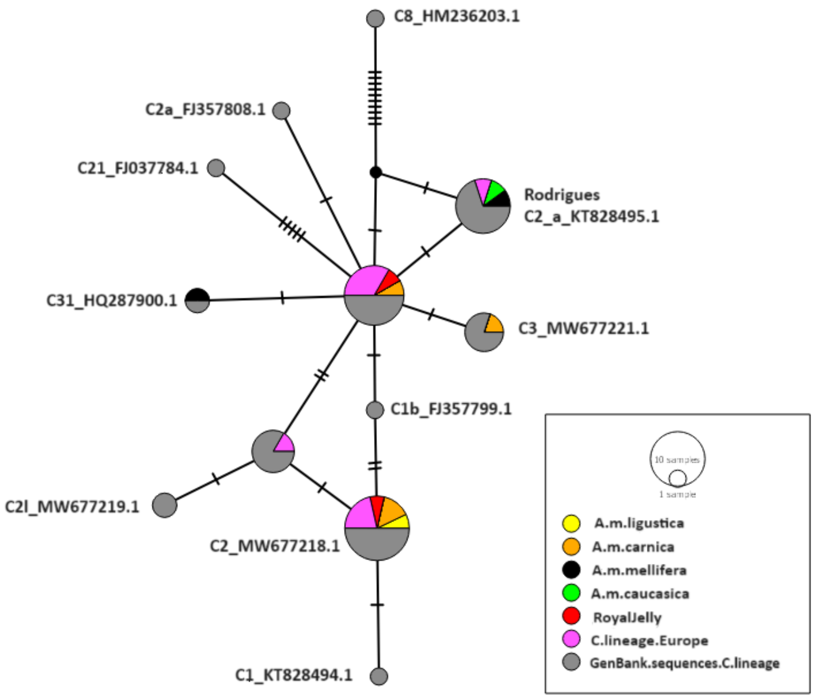

Figure S1. Median-joining network inferred from sequences from C lineage (Q), with the C.lineage.Europe sequences correspond to individuals sampled in Europe with no data reported on the sub-species name

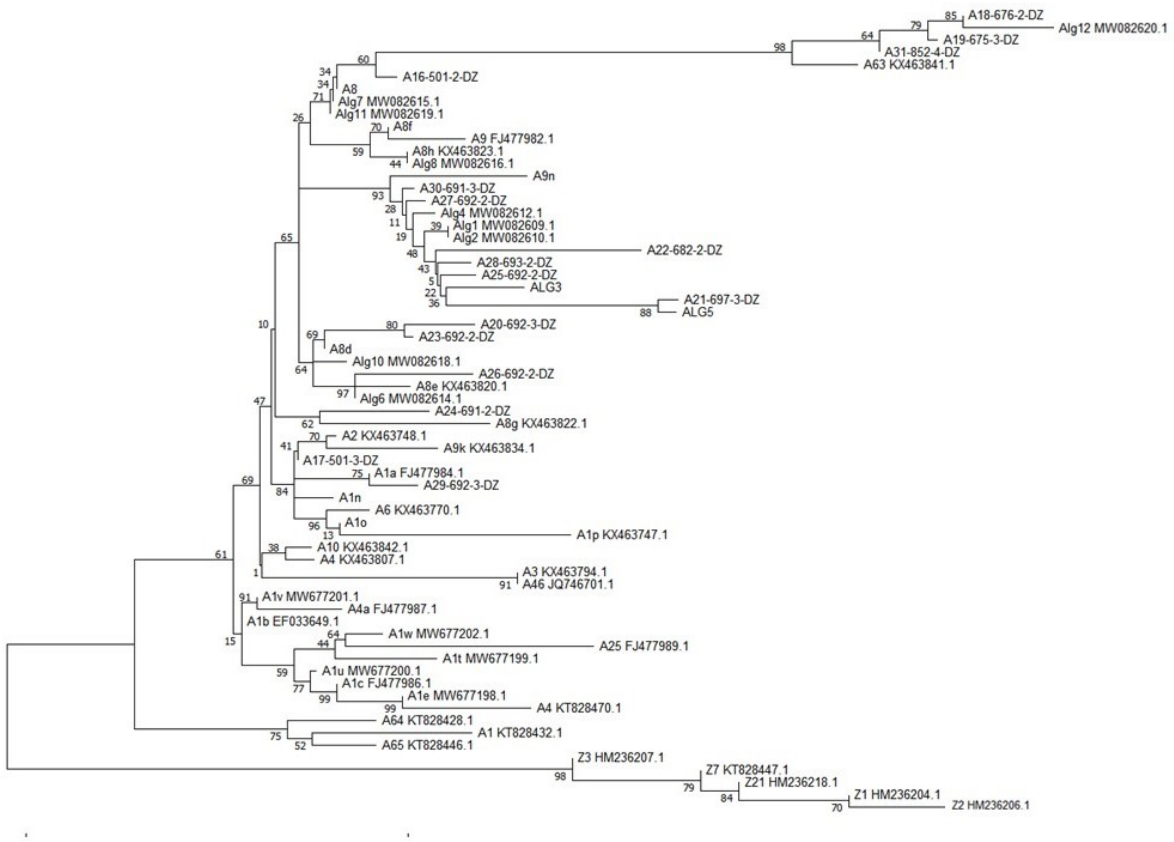

Figure S2. Phylogenetic tree of A lineage haplotypes. The tree is neighbor-joining. Nodes are supported by 1000 bootstraps
